# Reduced Radiation Exposure Protocol during Computer Tomography of the Left Atrium Prior to Catheter Ablation in Patients with Atrial Fibrillation

**DOI:** 10.3390/diagnostics12030612

**Published:** 2022-03-01

**Authors:** Tomasz Jadczyk, Jiri Wolf, Martin Pesl, Filip Soucek, Frantisek Lehar, Jiri Jez, Tomas Kulik, Bohdan Tyshchenko, Silvie Belaskova, Petr Ourednicek, Guido Caluori, Miroslav Novak, Zdenek Starek

**Affiliations:** 1 Interventional Cardiac Electrophysiology Group, International Clinical Research Center, St. Anne’s University Hospital Brno, 656 91 Brno, Czech Republic; tomasz.jadczyk@gmail.com (T.J.); jiri.wolf@philips.com (J.W.); martin.pesl@fnusa.cz (M.P.); filip.soucek@fnusa.cz (F.S.); frantisek.lehar@fnusa.cz (F.L.); jiri.jez@fnusa.cz (J.J.); kulikt@gmail.com (T.K.); guido.cal87@gmail.com (G.C.); 2 Department of Cardiology and Structural Heart Diseases, Medical University of Silesia, 40-635 Katowice, Poland; 3 1st Department of Internal Medicine—Cardioangiology, St. Anne’s University Hospital Brno, 656 91 Brno, Czech Republic; miroslav.novak@fnusa.cz; 4 Department of Biology, Faculty of Medicine, Masaryk University, 625 00 Brno, Czech Republic; 5 Biostatistics, International Clinical Research Center, St. Anne’s University Hospital Brno, 656 91 Brno, Czech Republic; tishchenko.b.m@gmail.com (B.T.); silvie.belaskova@fnusa.cz (S.B.); 6 Institute of Mathematics and Statistics, Masaryk University, 611 37 Brno, Czech Republic; petr.ourednicek@philips.com; 7 Department of Medical Imaging, Faculty of Medicine, St. Anne’s University Hospital Brno, Masaryk University, 656 91 Brno, Czech Republic; 8 Nanotechnology, CEITEC, Masaryk University, 625 00 Brno, Czech Republic; 9 IHU Liryc Electrophysiology and Heart Modeling Institute, Fondation Bordeaux Université, 33600 Pessac, France; 10 INSERM, UMR 1045, Cardiothoracic Research Center of Bordeaux, University Bordeaux, 33600 Pessac, France

**Keywords:** computed tomography, catheter ablation, radiation

## Abstract

(1) Background: Computer tomography (CT) is an imaging modality used in the pre-planning of radiofrequency catheter ablation (RFA) procedure in patients with cardiac arrhythmias. However, it is associated with a considerable ionizing radiation dose for patients. This study aims to develop and validate low-dose CT scanning protocols of the left atrium (LA) for RFA guidance. (2) Methods: 68 patients scheduled for RFA of atrial fibrillation were sequentially assigned to four groups of ECG-gated scanning protocols, based on the set tube current (TC): Group A (*n* = 20, TC = 33 mAs), Group B (*n* = 18, TC = 67 mAs), Group C (*n* = 10, TC = 135 mAs), and control Group D (*n* = 20, TC = 600 mAs). We used a 256-row multidetector CT with body weight-dependent tube voltage of 80 kVp (<70 kg), 100 kVp (70–90 kg), and 120 kVp (>90 kg). We evaluated scanning parameters including radiation dose, total scanning procedure time and signal-to-noise ratio (SNR). (3) Results: The average effective radiation dose (ED) was lower in Group A in comparison to Group B, C and D (0.83 (0.76–1.10), 1.55 (1.36–1.67), 2.91 (2.32–2.96) and 9.35 (8.00–10.04) mSv, *p* < 0.05). The total amount of contrast media was not significantly different between groups. The mean SNR was 6.5 (5.8–7.3), 7.1 (5.7–8.2), 10.8 (10.1–11.3), and 12.2 (9.9–15.7) for Group A, B, C and D, respectively. The comparisons of SNR in group A vs. B and C vs. D were without significant differences. (4) Conclusions: Optimized pre-ablation CT scanning protocols of the LA can reduce an average ED by 88.7%. Three dimensional (3D) models created with the lowest radiation protocol are useful for the integration of electro-anatomic-guided RFA procedures.

## 1. Introduction

Despite great technological progress in the management of patients with atrial fibrillation (AF), pulmonary vein isolation (PVI) with catheter ablation might be a challenging task even for an experienced operator. Structural variabilities of the left atrium (LA) and pulmonary veins (PVs) can include LA enlargement, thepresence of LA diverticulum, additional PVs with variant anatomy of the ostia (observed in 40% of patients undergoing ablation) [1], and early-branching [2,3]. Thus, it is convenient to support electroanatomic mapping (EAM)-guided radiofrequency ablation (RFA) procedures with additional imaging of the LA, such as 3D computed tomography (CT) [4,5,6,7]. Moreover, cardiac CT allows the assessment of LA appendage for the presence of thrombus further reducing the risk of complications [8]. However, despite the significant clinical usability of CT-generated LA models, this technique is associated with substantial ionizing radiation and contrast agent exposure for patients. Thus, new scanning protocols are required to improve patient safety.

We designed low-dose LA CT scanning protocols and validated them using a standard on-site hospital CT scanner, to show the feasibility and applicability in everyday clinical workflow. Furthermore, CT-generated LA models were co-registered with the EAM system for guidance of AF catheter ablation. We validated three prospectively gated axial protocols (group A–C) with standard on-site helical scan protocol as a control (group D, data collected retrospectively).

## 2. Materials and Methods

This ambispective single-center open study was designed to compare four pre-ablation CT scanning protocols of LA with regards to radiation dose, contrast media volume and total scanning procedure time. The CT-generated models were assessed qualitatively and semi-quantitatively by the operator’s opinion and evaluation of signal-to-noise ratio (SNR). The adequacy of the low-dose CT scanning protocol was estimated with: (1) application of a reduced effective radiation dose (ED); (2) appropriate 3D representation of LA and PV anatomy; and (3) possibility to integrate the CT model with EAM to support PVI procedures.

### 2.1. Study Population

Between October 2015 and May 2017, sixty-eight (68) patients with drug-resistant paroxysmal and persistent AF referred for catheter ablation were enrolled in the study. Exclusion criteria were: (1) allergy to iodine-containing contrast medium, (2) contraindication to anticoagulant therapy, (3) inability to follow instructions to hold one’s breath, (4) renal failure requiring dialysis or GFR < 30 mL/min/1.73 m^2^, (5) severe mitral regurgitation (to preserve homogenous contrast filling of LA), (6) LA thrombus, (7) pregnancy and (8) hemodynamic instability requiring intravenous inotropes.

During the prospective phase of the ambispective study, the individuals were grouped and underwent pre-ablation CT scanning of the LA according to one of the protocols designed with respect to the desired tube current (TC): Group A (TC = 33 mAs), Group B (TC = 67 mAs), Group C (TC = 135 mAs). In the retrospective phase of the ambispective study, we collected patients in control group D (TC = 600 mAs) from a hospital database as a scanning protocol reference routinely used at the Department of Radiology, International Clinical Research Center, St. Anne’s University Hospital Brno, Czech Republic (retrospective scanning). Detailed characterizations of each scanning protocol A–D are presented in Table S1.

### 2.2. Scanning Procedure

The examinations were performed on a 256-slice scanner (Brilliance iCT 256; Philips Healthcare, Best, The Netherlands). Scanning protocols differed regarding body weight-dependent tube voltage and tube current with collimation automatically adapted to the length of the scan area. Prospective scans A–C applied half of the full rotation angle needed for data collection and not used overlapping angle in the time of diastole of cardiac cycle. In retrospective helical protocol D, the pitch factor was automatically calculated based on the patient’s heart rate. Additionally, multicycle technology was used to reach maximum temporal resolution. Specifically, all CT scanning protocols were subdivided into three sections, based on patient weight, to allow the appropriate quality of imaging. Peak tube voltages of 80 kVp, 100 kVp, and 120 kVp were adapted according to patient body weight categories of <70 kg, 70–90 kg, and >90 kg, respectively. Tube time-current product (in mAs) was the same across the three sections of each scanning protocol and defined the inclusion of patients to the relative study group. All images were reconstructed with the same level of hybrid iterative reconstruction (hIR, iDose4, level 7). A detailed characterizations of each parameter are presented in Table S1. In the axial scan, slice thickness was 0.625 mm. All prospective scans were conducted with zero tolerance to avoid additional doses. Planned CT dose index (CTDI) is shown in Table S1. In this study, we have not used any other dose modulation techniques to further decrease dose radiation. To determine an exact field of interest (FOI) for contrast scan, we performed ultralow dose native prospective calcium scoring scan in each patient—weight-adjusted scans parameters of 120 kVp and a minimum tube current of 10 mAs, Table S1. The dose length product (DLP) of the LA and PV scans were recorded from CT scanner display or dose report. The effective radiation dose was calculated using the equation:

ED = kDLP,(1)

where k = 0.017 mSv × mGy^−1^ × cm^−1^ for the cardiac CT [9].

**Figure 1:**
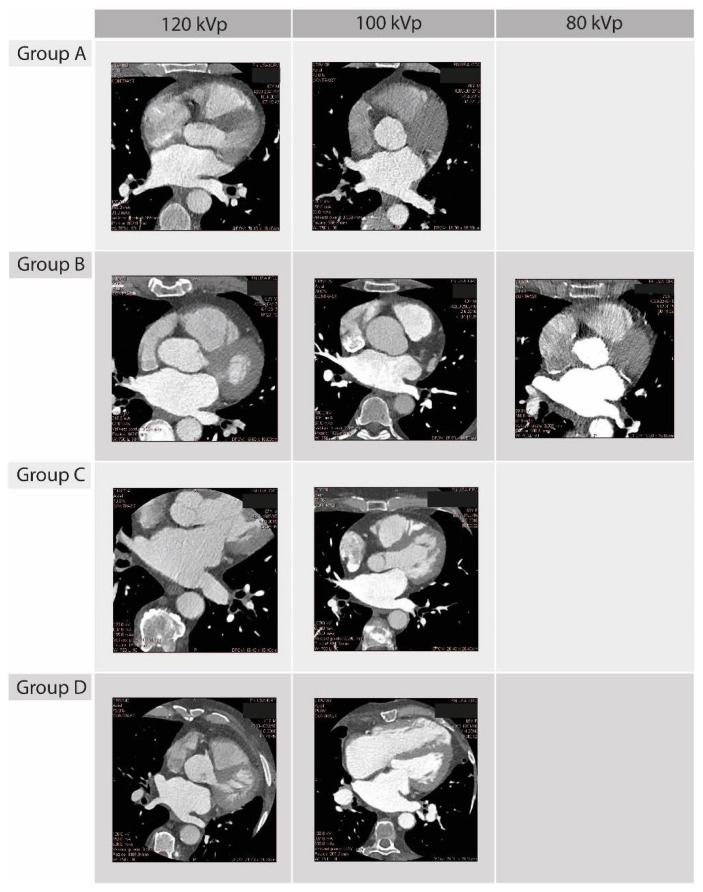
Examples of LA images performed using Group A–D scanning protocols. A 256-row multidetector CT with body weight-dependent tube voltage of 80 kVp (<70 kg), 100 kVp (70–90 kg), and 120 kVp (>90 kg) was used with tube current of 33 mAs, 67 mAs, 135 mAs and 600 mAs for Group A–D, respectively.

Figure 1 shows examples of LA images performed using Group A–D scanning protocols.

### 2.3. Pre-Scanning Settings

**Figure 2:**
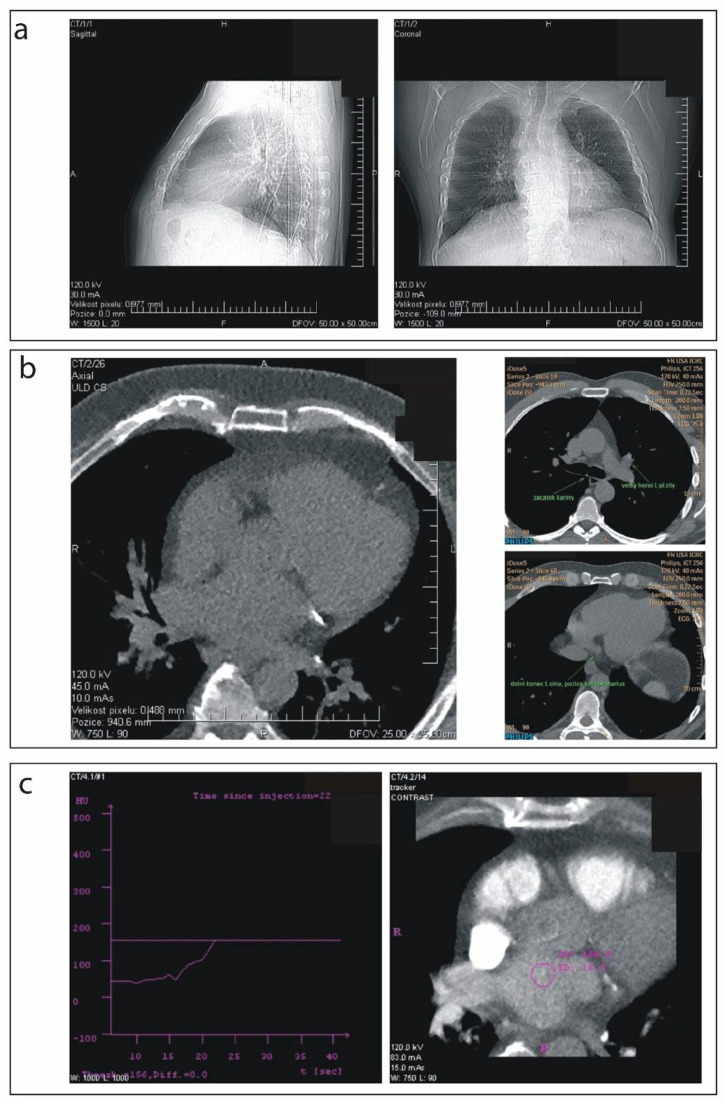
(**a**) Dual surview (scout in the two cross-sections); (**b**) ultra-low-dose calcium scoring; (**c**) setting of contrast agent tracker.

Patients were instructed to maintain a normal breathing cycle throughout the study but were asked to follow the operator’s verbal commands for breath-hold during active CT scanning. All patients were imaged in a supine position with their arms raised above their heads. Isocentering of the LA was obtained from the anteroposterior and left lateral X-ray views. In all protocols, the target area of interest (LA and surroundings) was precisely located before the start of the scanning procedure by using a standard dual CT radiograph (a surview) (Figure 2a) and ultralow-dose calcium scoring (Figure 2b). After precise focusing on an LA, a contrast agent tracker was placed in the middle of the LA (Figure 2c) and data acquisition was launched. The total amount of Iomeron 400 contrast (Bracco Imaging S.p.A., Milano, Italy) was calculated individually according to patient weight (0.75 mL/kg). Contrast agent was administered into peripheral veins using the angiographic injection system (Mark-V ProVis, Medrad, Inc., Indianola, PA, USA) with the following modality: (i) 50 mL of contrast agent was injected at 5 mL/s rate; (ii) the rest of the contrast agent was injected at 4 mL/s; (iii) 50 mL of saline was injected at 5 mL/s. The start of the scanning phase was triggered by the tracker threshold set to 110 Hounsfield units (HU) (Figure 2c).

### 2.4. 3D Left Atrium Model Segmentation and Image Quality Assessment

EP Navigator^®^ 3D image integration tool (Philips Healthcare, Hamburg, Germany) was used to segment CT data. Images were post-processed by an electrophysiology technician with 10 years of experience in cardiac CT analysis. The computer-derived 3D model of the LA was evaluated by two independent electrophysiologists who had over 8 years of cardiac CT reading experience. Image quality was semi-quantitatively categorized using a 3-point Likert scale: (1) excellent—LA contour and all PVs were recognized and segmented automatically; (2) useful—LA contour recognized but some of PVs needed to be segmented manually; and (3) inadequate—LA contour and/or PVs were not recognized properly, 3D reconstruction was not feasible or the details were not interpretable [10]. After the segmentation process, both electrophysiologists evaluated LA models individually. Grading was concordant (agreement) if both physicians gave the same mark.

Quantitative assessment was performed based on the intraluminal attenuation (IA) with standard deviation (noise) in the LA. The same measurement position was chosen in all three planes (frontal, sagittal, transversal) with circular ROIs (1 cm^2^) placed in the center of LA. SNR was calculated as [11]:

SNR = IA/noise.(2)

### 2.5. CT-EAM Integration and Procedural Usefulness

The resulting LA models were integrated with the electroanatomical map using the EnSite Verismo Segmentation Tool, a component of the EnSite Velocity cardiac mapping system (Abbott Laboratories, Chicago, IL, USA). Integration was considered successful if the LA model was imported to the EAM system without errors and the operator confirmed the completeness of the import process. Procedural usefulness was assessed by the electrophysiologist performing the PVI using dichotomous categorization to (1) Useful or (2) Not useful.

### 2.6. Statistical Analysis

The Shapiro–Wilk test was used to assess the normal distribution of data. For the normal distribution, data were analyzed by ANOVA, followed by the Scheffe test for comparison of samples, and are presented as mean ± standard deviations (SD). The Kruskal–Wallis test, followed by Dunn’s multiple comparison test (Benjamini–Hochberg-corrected *p*-value < 0.05), was applied for ordinal data, which is presented as medians (25th–75th IQR). A generalized linear model (GLM) for binomial response variable and logit link function was computed to predict software successful integration (categorical variable) in relation to different protocols tested in the present study. Statistical analysis was carried out using SAS (Copyright © 2017, SAS Institute Inc., Cary, NC, USA).

## 3. Results

Baseline patient characteristics are presented in Table 1. The median age of the study population was 61.0 (53.3–68.0) years with a numerically higher number of males among all participants (79.6%). The median body mass index (BMI) of all participants was 30.8 (26.8–32.6). Between the groups, there were no statistically significant differences in age, sex, or BMI. The four groups were homogeneous in terms of cardiovascular risk factors. RFA procedures were successful in all patients. No procedural complications were noted during CT scanning and intervention. During CT scanning, sinus rhythm was present in 32 patients and AF in 36 individuals. There was no statistically significant difference in heart rhythm distribution between groups (*p* = 0.241).

### 3.1. Dose Results

Data acquisition by 256-row MDCT was successful in all 68 patients (100%). The ECG-gated prospective axial protocol with 33 mAs tube current (Group A) allowed reduction of DLP and corresponding ED to 48.6 (44.5–64.8) mGy × cm and 0.83 (0.76–1.10) mSv, respectively, with the lowest ED value of 0.83 mSv using 80 kVp tube voltage. In comparison, radiation doses in Groups B, C and D were significantly higher at 1.55 (1.36–1.67), 2.91 (2.32–2.96) and 9.35 (8.00–10.04) mSv, respectively). The total scanning procedure time and amount of contrast media were not statistically different among the groups; details are presented in Table 2.

### 3.2. Quality of 3D Left Atrium Models, CT-EAM Integration and Procedural Usefulness

Successful image segmentation and reconstruction were achieved in all cases. For all 68 LA scans, there was full agreement in image quality scoring between both electrophysiologists. Overall, LA 3D models were graded excellent in 53 cases (77.94%) and useful in 15 cases (22.06%), with eight requiring minimal manual segmentation (up to 8 min for the presence of three right PVs), while four cases required up to 15 min of manual processing (due to contrast presence in the right atrium). No model was found to be inadequate.

Specifically for Group A, which presented the lowest radiation dose, 14 of 20 scans (70%) were graded excellent, and only one required advanced manual processing as defined above. Furthermore, heart rhythm did not affect the 3D reconstruction process and final model quality. There were no statistically significant differences between scanning protocols in terms of integration of CT images with the EAM system (*p* = 0.296).

In our GLM of image integration into the EAM software, Group D was considered as a control, and odds ratios estimated across the comparisons were Group C vs. Group D protocol (OR = 0.123; 95% CI 0.011–1386; *p* = 0.6320), Group B vs. Group D protocol (OR = 0.137; 95% CI 0.014–1.311; *p* = 0.3904) and Group A vs. Group D protocol (OR = 0.123; 95% CI 0.013–1.138; *p* = 0.267. Other models were considered with adjusted effects for BMI, cardiac rhythm and age, yet none of these additional parameters made any significant change in the test. The odds ratio estimated were: A vs. D protocol OR = 0.408; 95% CI 0.034–4.852 with *p* = 0.789; B protocol vs. D protocol OR = 0.423; 95% CI 0.035–5.058 with *p* = 0.843; C protocol vs. D protocol is OR = 0.286; 95% CI 0.019–4.284 with *p* = 0.450.

**Figure 3:**
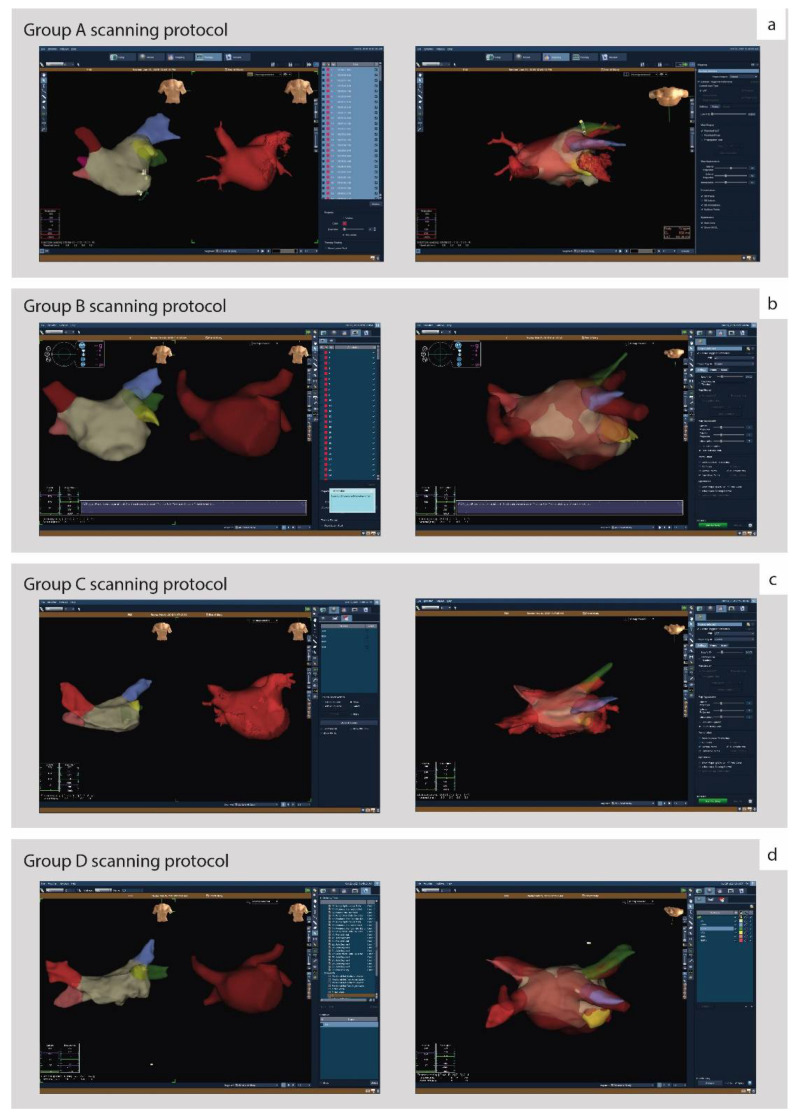
Parallel views of 3D electroanatomical maps with CT-generated LA models. From left to right, each subfigure represents the patient’s electroanatomical maps (beige with PVs colorized), the corresponding CT LA model (red) and the fusion of the two. (**a**) Group A (current tube 33 mAs); (**b**) Group B (current tube 67 mAs); (**c**) Group C (current tube 135 mAs); (**d**) Group D (current tube 600 mAs).

The resulting 3D LA models were used to support the EAM procedure either as a parallel view of the electroanatomical map and 3D model of LA (Figure 3a–d, left panels) or as a direct fusion of the 3D LA model with the electroanatomical map (Figure 3a–d, right panels). Both methods provided additional information about complex LA anatomy for electrophysiologists. Anatomical accuracy was reassessed through the whole procedure, and no discordance was reported. Based on the operator’s opinion, all CT-generated models were procedurally useful and supportive for RFA including 12 cases (17.64%) requiring additional manual segmentation.

Among all scanning protocols, the SNR was the highest in Group D (12.2 (9.9–15.7) reaching statistical significance in comparison to Group A (6.5 (5.8–7.3)) and Group B (7.1 (5.7–8.2), *p* <0.0001 Mean SNR was, 7.1 (5.7–8.2), 10.8 (10.1–11.3), and) for Groups A, B, C, and D, respectively (*p* < 0.0001 for group A vs. C and B vs. C). The comparisons of SNR in group A vs. B and C vs. D were without significant difference. Moreover, there was no statistically significant difference between standard hospital scanning protocols applied in Group D and C protocols. SNR, IA, and noise values are presented in Table 2.

## 4. Discussion

The rapid growth of AF prevalence in the elderly European population indicates there will be an estimated ≥14.4 million patients affected by this arrhythmia by the year 2060 [12]. The present demographic scenario is reflected by the number of individuals referred to PVI by EAM-guided catheter ablation [13]. Compensating for LA/PVs anatomical variability [14], which might reduce therapy efficacy and increase procedural time, different imaging modalities have been used to facilitate RFA procedures [4,15,16,17] including CT. Currently, CT scanners with excellent spatial resolution are widely distributed and could be helpful for detailed pre-procedural planning in cardiac electrophysiology. However, despite its great clinical utility [5,18], this imaging technique is associated with substantial radiation and potential long-term cancer risks due to ionizing radiation [19]. Thus, in the present study, we described low-dose CT scanning protocols that can be implemented on the standard on-site hospital CT scanner for pre-RFA imaging of LA and EAM-CT co-registration and fusion. Previously, various CT protocols have been developed, including non-ECG gated scanning, retrospective and prospective ECG-triggered axial scanning (Step & Shoot Cardiac) [20,21], dual-source high-pitch spiral scanning [22,23], adaptive statistical iterative reconstruction algorithm (ASIR; GE Healthcare, Milwaukee, WI, USA) and model-based iterative reconstruction (MBIR) technology [24]. The ECG-gated 64-row CT generates a relatively high radiation dose (13.4 mSv) requiring substantial contrast volume for adequate LA image quality. In comparison, non-ECG gated 64-row CT allows the reduction of the radiation dose to 4.6 mSv [20]. Furthermore, Annoni et al. used the MBIR algorithm to improve image quality with ED reduction to 0.4 mSv. However, applied technology required high computing power and a long reconstruction time [24]. In our study, comparing to the results reported by Annoni et al., calcium score imaging accounted for 29% of the ED and was implemented to plan FOI precisely. Importantly, there is a chance to further reduce ED by omitting this step, while determining FOI from a dual surview scan. Moreover, we used a co-efficient factor k = 0.017 mSv × mGy^−1^ × cm^−1^, while Annoni et al. [24] and Fahlenkamp et al. [11] applied k = 0.014 mSv × mGy^−1^ × cm^−1^. As computationally modeled, for a patient with the lowest DLP achieved in our study, we could reduce ED to 0.31 mSv (k = 0.014 mSv × mGy^−1^ × cm^−1^). Notably, further optimization of the scanning protocol can be achieved using dedicated software for mathematical modeling of the lowest SNR to assess noise threshold acceptable by 3D reconstruction workstation, which is a goal for a further investigation. A merger of CT-reconstructed 3D models of the LA with the EAM system is an excellent supportive tool providing anatomic orientation in real-time. In our study, a fusion of the 3D LA model with the EnSite Velocity was possible in all cases. After registration of the 3D CT image to the EAM map, the LA models were consistently continuously oriented at the same angles the electroanatomical reconstruction. Moreover, the 3D image could be clipped manually for independent internal view assessment.

The resulting CT images provided valuable anatomical information enabling favorable logistics and seamless utilization in the electrophysiology laboratory. Optimization of the LA CT scanning protocol is a promising approach for reducing patients’ radiation exposure as well as increasing therapeutic efficacy due to better anatomical orientation during mapping and ablation. Primarily, the present study has a practical aspect, supporting the better utilization of available on-site hospital resources for workflow improvement and sustaining the quality of diagnostic and interventional electrophysiology procedures. 

It is worth noting that the application of cardiac CT expends beyond supporting ablation procedures. This imaging tool is used in clinical settings to diagnose procedural complications (i.e., LA dissection) [25], confirm/exclude the presence of thrombus [8] and plan structural heart disease procedures [26]. Parallelly, cardiac CT can be used to quantify coronary calcification burden stratifying asymptomatic patients with low and intermediate risk of cardiovascular events [27]. In this aspect, the expert review on this imaging technique highlights its predictive value beyond the traditional Framingham risk score [28]. The aforementioned examples confirm a wide range of clinical applicability and rationalize the broader clinical utilization of cardiac CT.

Limitations: This is a single-center study with only one imaging modality evaluated. An additional comparison with 3D rotational angiography, cardiac magnetic resonance, and intracardiac echocardiography would provide a more detailed understanding of a preferable diagnostic strategy. Despite detailed characteristics of low-dose scanning protocols, neither acute procedural data nor follow-up data were presented. Therefore, no conclusion on mid- and long-term outcomes can be made. Due to the relatively small number of patients enrolled in the study, larger, multicenter clinical trials are needed to confirm the promising presented findings.

## 5. Conclusions

Optimized left atrium CT scan protocols using hIR allow the creation of quality 3D models of the LA with a reduction in the average ED by 86.2%. Newly developed scanning protocols are simple to implement and could be applied to the standard CT workstation. Low-dose CT-generated 3D models are non-inferior to routinely used models in terms of usefulness for 3D EAM integration and guidance during RFA procedures.

## Figures and Tables

**Table 1:** Patient characteristics.

	Group A (*n* = 20)	Group B (*n* = 18)	Group C (*n* = 10)	Group D (*n* = 20)	*p*-Value
Age (years)	61.0 (53.0–68.0)	62.0 (56.0–66.0)	56.0 (49.0–68.0)	63.0 (58.0–69.0)	0.333
Male, *n* (%)	17 (85)	16 (89)	7 (70)	14 (70)	0.392
BMI (kg/m^2^)	28.0 (26.4–33.8)	30.6 (26.3–34.0)	31.1 (28.1–32.0)	30.0 (27.7–32.2)	0.947
Diabetes mellitus (yes), *n* (%)	6 (30)	4 (22)	1 (10)	2 (10)	0.354
Hypertension (yes), *n* (%)	12 (60)	12 (67)	4 (40)	11 (55)	0.578
Dyslipidemia (yes), *n* (%)	10 (50)	6 (33)	6 (60)	8 (40)	0.514
Ischemic heart diseases (yes), *n* (%)	3 (15)	4 (22)	1 (10)	3 (15)	0.848
TEE, LA diameter (mm)	54.0 (46.0–60.0)	48.5 (45.0–52.5)	52.2 (47.0–58.0)	46 (40.0–55.0)	0.227
Preimaging heart rhythm, SR, *n* (%)	8 (40)	8 (44)	3 (30)	13 (65)	0.241

AF = atrial fibrillation; BMI = body mass index; LA = left atrium; SR = sinus rhythm; TEE = trans-esophageal echocardiography Data presented as median (25th; 75th IQR) or percentage (%).

**Table 2:** CT procedure characteristics.

	Group A (*n* = 20)	Group B (*n* = 18)	Group C (*n* = 10)	Group D (*n* = 20)	*p*-Value					
					A vs. B	A vs. C	A vs. D	B vs. C	B vs. D	C vs. D
DLP (mGy × cm)	48.6 (44.5–64.8)	90.9 (79.8–98.1)	171.2 (131.4–174.2)	550.2 (470.7–590.7)	<0.0001	<0.0001	<0.0001	<0.0001	<0.0001	<0.0001
Effective radiation dose (mSv) ^§^	0.83 (0.76–1.10)	1.55 (1.36–1.67)	2.91 (2.23–2.96)	9.35 (8.00–10.04)	<0.0001	<0.0001	<0.0001	<0.0001	<0.0001	<0.0001
Total scanning procedure time (min) ^#^	8.0 (7.0–11.5)	7.0 (7.0–9.0)	8.0 (7.0–11.9)	10.0 (8.0–11.0)	NS.	NS	<0.0001	NS	<0.0001	0.003
Contrast media volume (ml) ^#^	66.5 (60.0–78.5)	70.5 (64.0–75.0)	76.0 (67.0–80.0)	100.0 (80.0–100.0)	NS	NS	NS	NS	NS	NS
IA ± noise (HU)	386.5 (317.2–469.6)	370.1 (280.4–451.3)	378.0 (332.0–424.0)	341.2 (306.9–97.9)	NS	NS	NS	NS	NS	NS
SNR	6.5 (5.8–7.3)	7.1 (5.7–8.2)	10.8 (10.1–11.3)	12.2 (9.9–15.7)	NS	<0.001	<0.0001	0.0004	<0.0001	n.s.

IA = intraluminal attenuation; CT = computer tomography; DLP = dose length product; HU—Hounsfield unit; SNR = signal-to-noise ratio; NS. = not significant; Data presented as median (25th–75th IQR); ^§^ conversion coefficient factor k = 0.017 mSv × mGy^−1^ × cm^−1^; ^#^ Kruskal-Wallis test not statistically significant.

## Supplementary Materials

The following supporting information can be downloaded at: https://www.mdpi.com/article/10.3390/diagnostics12030612/s1, Table S1: CT scanning protocols.

## References

[1] Kato R., Lickfett L., Meininger G., Dickfeld T., Wu R., Juang G., Angkeow P., LaCorte J., Bluemke D., Berger R. (2003). Pulmonary vein anatomy in patients undergoing catheter ablation of atrial fibrillation: Lessons learned by use of magnetic resonance imaging. Circulation.

[2] Chen J., Yang Z.-G., Xu H.-Y., Shi K., Long Q.-H., Guo Y.-K. (2017). Assessments of pulmonary vein and left atrial anatomical variants in atrial fibrillation patients for catheter ablation with cardiac CT. Eur. Radiol..

[3] Skowerski M., Wozniak-Skowerska I., Hoffmann A., Nowak S., Skowerski T., Sosnowski M., Wnuk-Wojnar A.M., Mizia-Stec K. (2018). Pulmonary vein anatomy variants as a biomarker of atrial fi-brillation—CT angiography evaluation. BMC Cardiovasc. Disord..

[4] Anand R., Gorev M., Poghosyan H., Pothier L., Matkins J., Kotler G., Moroz S., Armstrong J., Nemtsov S.V., Orlov M.V. (2016). Prospective randomized comparison of rotational angiography with three-dimensional reconstruction and computed tomography merged with electro-anatomical mapping: A two center atrial fibrillation ablation study. J. Interv. Card. Electrophysiol..

[5] Dong J., Calkins H., Solomon S.B., Lai S., Dalal D., Lardo A., Brem E., Preiss A., Berger R.D., Halperin H. (2006). Integrated electroanatomic mapping with three-dimensional computed tomo-graphic images for real-time guided ablations. Circulation.

[6] Kistler P., Rajappan K., Harris S., Earley M.J., Richmond L., Sporton S.C., Schilling R.J. (2008). The impact of image integration on catheter ablation of atrial fibrillation using electroanatomic mapping: A prospective randomized study. Eur. Heart J..

[7] Richmond L., Rajappan K., Voth E., Rangavajhala V., Earley M.J., Thomas G., Harris S., Sporton S.C., Schilling R.J. (2008). Validation of Computed Tomography Image Integration into the EnSite NavX Mapping System to Perform Catheter Ablation of Atrial Fibrillation. J. Cardiovasc. Electrophysiol..

[8] Park J.J. (2019). Computed Tomography for Assessment of Left Atrial Appendage Function. Korean Circ. J..

[9] Protection R. (2007). The 2007 Recommendations of the International Commission on Radiological Protection. ICRP Publication 103. Ann. ICRP.

[10] Wolf J., Stárek Z., Jež J., Lehar F., Lukasova M., Kulik T., Novak M. (2015). Rotational angiography of left ventricle to guide ventricular tachycardia ablation. Int. J. Cardiovasc. Imaging.

[11] Fahlenkamp U.L., Diaz Ramirez I., Wagner M., Schwenke C., Huppertz A., Hamm B., Lembcke A. (2018). Image quality of low-radiation dose left atrial CT using filtered back projection and an iterative reconstruction algorithm: Intra-individual comparison in unselected patients undergoing pul-monary vein isolation. Acta Radiol..

[12] Di Carlo A., Bellino L., Consoli D., Mori F., Zaninelli A., Baldereschi M., Cattarinussi A., D’Alfonso M.G., Gradia C., Sgherzi B. (2019). Prevalence of atrial fibrillation in the Italian elderly population and projections from 2020 to 2060 for Italy and the European Union: The FAI Project. EP Eur..

[13] Kirchhof P., Benussi S., Kotecha D., Ahlsson A., Atar D., Casadei B., Castella M., Diener H.C., Heidbuchel H., Hendriks J. (2016). 2016 ESC Guidelines for the management of atrial fibrillation developed in col-laboration with EACTS. Eur. Heart J..

[14] Hunter R.J., Ginks M., Ang R., Diab I., Goromonzi F.C., Page S., Baker V., Richmond L., Tayebjee M., Sporton S. (2010). Impact of variant pulmonary vein anatomy and image integration on long-term out-come after catheter ablation for atrial fibrillation. Europace.

[15] Malchano Z.J., Neuzil P., Cury R.C., Holmvang G., Weichet J., Schmidt E.J., Ruskin J.N., Reddy V.Y. (2006). Integration of Cardiac CT/MR Imaging with Three-Dimensional Electroanatomical Mapping to Guide Catheter Manipulation in the Left Atrium: Implications for Catheter Ablation of Atrial Fibrillation. J. Cardiovasc. Electrophysiol..

[16] Mah D.Y., Miyake C.Y., Sherwin E.D., Walsh A., Anderson M.J., Western K., Abrams D., Alexander M.E., Cecchin F., Walsh E.P. (2013). The use of an integrated electroanatomic mapping system and intracardiac echocardiography to reduce radiation exposure in children and young adults undergoing ablation of supraventricular tachycardia. Europace.

[17] Stárek Z., Lehar F., Jež J., Wolf J., Novak M. (2014). 3D X-ray imaging methods in support catheter ablations of cardiac arrhythmias. Int. J. Cardiovasc. Imaging.

[18] Tian J., Jeudy J., Smith M.F., Jimenez A., Yin X., Bruce P.A., Lei P., Turgeman A., Abbo A., Shekhar R. (2010). Three-Dimensional Contrast-Enhanced Multidetector CT for Anatomic, Dynamic, and Perfusion Characterization of Abnormal Myocardium To Guide Ventricular Tachycardia Ablations. Circ. Arrhythmia Electrophysiol..

[19] Picano E., Vano E. (2011). The Radiation Issue in Cardiology: The time for action is now. Cardiovasc. Ultrasound.

[20] Wagner M., Butler C., Rief M., Beling M., Durmus T., Huppertz A., Voigt A., Baumann G., Hamm B., Lembcke A. (2010). Comparison of non-gated vs. electrocardiogram-gated 64-detector-row computed tomography for integrated electroanatomic mapping in patients undergoing pulmonary vein isolation. Europace.

[21] Hlaihel C., Boussel L., Cochet H., Roch J.A., Coulon P., Walker M.J., Douek P.C. (2011). Dose and image quality comparison between prospectively gated axial and retro-spectively gated helical coronary CT angiography. Br. J. Radiol..

[22] Thai W.-E., Wai B., Lin K., Cheng T., Heist E.K., Hoffmann U., Singh J.P., Truong Q.A. (2012). Pulmonary Venous Anatomy Imaging with Low-Dose, Prospectively ECG-Triggered, High-Pitch 128-Slice Dual-Source Computed Tomography. Circ. Arrhythmia Electrophysiol..

[23] Iwayama T., Arimoto T., Ishigaki D., Hashimoto N., Kumagai Y., Koyama Y., Kiribayashi N., Netsu S., Nishiyama S., Takahashi H. (2015). The Clinical Value of Nongated Dual-Source Computed Tomography in Atrial Fibrillation Catheter Ablation. J. Cardiovasc. Electrophysiol..

[24] Annoni A.D., Andreini D., Pontone G., Formenti A., Petullà M., Consiglio E., Nobili E., Baggiano A., Conte E., Mushtaq S. (2015). Ultra-low-dose CT for left atrium and pulmonary veins imaging using new model-based iterative reconstruction algorithm. Eur. Heart J.—Cardiovasc. Imaging.

[25] Cereda A.F., de Luca F., Lanzone A.M., Cottini M., Pastori L., Sangiorgi G. (2020). Case report and systematic review of iatrogenic left atrial dissection in different cardiovascular specialties: A common treatment for an uncommon complication?. Catheter. Cardiovasc. Interv..

[26] Thériault-Lauzier P., Spaziano M., Vaquerizo B., Buithieu J., Martucci G., Piazza N. (2015). Computed Tomography for Structural Heart Disease and Interventions. Interv. Cardiol..

[27] Oudkerk M., Stillman A.E., Halliburton S.S., Kalender W.A., Möhlenkamp S., McCollough C.H., Vliegenthart R., Shaw L.J., Stanford W., Taylor A.J. (2008). Coronary artery calcium screening: Current status and recommendations from the European Society of Cardiac Radiology and North American Society for Cardiovascular Imaging. Int. J. Cardiovasc. Imaging.

[28] Hecht H.S. (2015). Coronary artery calcium scanning: Past, present, and future. JACC Cardiovasc. Imaging.

