# Supplementary material for: Reduced Radiation Exposure Protocol during Computer Tomography of the Left Atrium Prior to Catheter Ablation in Patients with Atrial Fibrillation
Source: Diagnostics (Basel). 2022 Mar 1;12(3):612. doi: 10.3390/diagnostics12030612 (PMC8947727; doi:10.3390/diagnostics12030612)
Supplement: Supplementary file 1 [file diagnostics-12-00612-s001.zip › diagnostics-1580889-supplementary.pdf]

diagnostics MDPI

Table S1. CT scanning protocols

Group	Gating type	Patient weight (kg)	kVp	mAs	Pitch /Steps	Phase (%)	RT (s)	Recon filter	Planned CTDI (mGy)	Planned DLP (mGy x cm-1)	CS protocol (kVp/mAs)	CS protocol planned CTDI/DLP (mGy-mGy x cm-1)	iDOse4 factor	Phase (%)
A	P	<70	80	33	0/2	78	0.27	XCB	0.70	10	120/10	0.8/10.8	7	78
	P	70-90	100	33	0/2	78	0.27	XCB	1.40	20	120/10	0.8/10.8	7	78
	P	>90	120	33	0/2	78	0.27	XCB	2.40	34	120/10	0.8/10.8	7	78
B	P	<70	80	67	0/2	78	0.27	XCB	1.40	20	120/10	0.8/10.8	7	78
	P	70-90	100	67	0/2	78	0.27	XCB	2.90	41	120/10	0.8/10.8	7	78
	P	>90	120	67	0/2	78	0.27	XCB	4.80	69	120/10	0.8/10.8	7	78
C	P	<70	80	135	0/2	78	0.27	XCB	2.80	40	120/10	0.8/10.8	7	78
	P	70-90	100	135	0/2	78	0.27	XCB	5.8	83	120/10	0.8/10.8	7	78
	P	>90	120	135	0/2	78	0.27	XCB	9.7	139	120/10	0.8/10.8	7	78
D	R	<70	80	600	0.18/0	75	0.27	XCB	11.1	208	120/25	1.9/27	6	75,50,25,0
	R	70-90	100	600	0.18/0	75	0.27	XCB	23	434	120/30	2.3/32	6	75,50,25,0
	R	>90	120	600	0.18/0	75	0.27	XCB	38.6	727	120/40	3.1/43.1	6	75,50,25,0

CS protocol = calcium scoring protocol; CS CTDI /DLP = corresponding planned CT dose index and dose length product for CS scan; CTDI = computer tomography dose index; DLP = dose length product; iDOse4 factor = selectable level (7 is maximum for heart region); kVp = tube kilovoltage peak; mAs = milliampere-seconds, tube time current product; Pitch/Steps = Value for retrospective helical (Pitch) and prospective gating (Steps) technique; P = Prospective gating; Phase = percent of reconstruction within RR interval; mGy = milligray R = retrospective gating; Recon filter = kernel reconstruction filter; RT = rotation time.
